# Effects of a Multicomponent Preventive Intervention in Women at Risk of Sarcopenia: A Pilot Study

**DOI:** 10.3390/healthcare12121191

**Published:** 2024-06-13

**Authors:** Violeta Rios-Escalante, Juan Carlos Perez-Barba, Maria Claudia Espinel-Bermudez, Ana Bertha Zavalza-Gomez, Elva Dolores Arias-Merino, Maria G. Zavala-Cerna, Sergio Sanchez-Garcia, Xochitl Trujillo, Arnulfo Hernan Nava-Zavala

**Affiliations:** 1 Departamento de Salud Publica, Centro Universitario de Ciencias de la Salud, Universidad de Guadalajara, Guadalajara 44340, Jalisco, Mexico; violetarios_e@hotmail.com (V.R.-E.); elva.arias@academicos.udg.mx (E.D.A.-M.); 2 Facultad de Medicina, Universidad de Colima, Colima 28040, Colima, Mexico; 3 Hospital de Ginecologia y Obstetricia, Centro Medico Nacional de Occidente, Instituto Mexicano del Seguro Social, Guadalajara 44329, Jalisco, Mexico; perezbarbadr@hotmail.com (J.C.P.-B.); ana.zavalza@imss.gob.mx (A.B.Z.-G.); 4 Unidad de Investigacion Biomedica 02, Hospital de Especialidades, Centro Medico Nacional de Occidente, Instituto Mexicano del Seguro Social, Guadalajara 44329, Jalisco, Mexico; 5 Unidad Academica Ciencias de la Salud, Universidad Autónoma de Guadalajara, Zapopan 45129, Jalisco, Mexico; maria.cerna@edu.uag.mx; 6 Unidad de Investigación Epidemiológica y en Servicios de Salud, Area Envejecimiento, Centro Medico Nacional Siglo XXI, Instituto Mexicano del Seguro Social, Ciudad de Mexico 06720, Mexico; sergio.sanchezga@imss.gob.mx; 7 Centro Universitario de Investigaciones Biomedicas, Universidad de Colima, Colima 28040, Colima, Mexico; rosio@ucol.mx; 8 Unidad de Investigacion Epidemiologica y en Servicios de Salud, Centro Medico Nacional de Occidente Organo de Operación Administrativa Desconcentrada Jalisco, Instituto Mexicano del Seguro Social, Guadalajara 44329, Jalisco, Mexico; 9 Programa Internacional de Medicina, Universidad Autónoma de Guadalajara, Zapopan 45129, Jalisco, Mexico; 10 Departamento de Inmunologia y Reumatologia del Hospital General de Occidente, Secretaria de Salud Jalisco, Guadalajara 45170, Jalisco, Mexico

**Keywords:** sarcopenia, multicomponent, aging, preventive

## Abstract

Sarcopenia is defined by the presence of decreased skeletal muscle mass, strength, and functionality in older people. Multicomponent interventions represent an alternative to non-pharmacological treatment for preventing disease progression. This study aimed to evaluate the effects of a multicomponent intervention approach in women at risk of sarcopenia. Methods: A quasi-experimental pilot study of 12 weeks was conducted, with 24 sessions of dancing and resistance exercises and 12 sessions of nutritional education. The outcomes were changes in muscle mass, grip strength, gait speed, and body composition. The project was registered on Clinical Trials: NCT06038500 (14 September 2023). Results: Twelve women aged 55–75 years participated in this study; after the intervention, changes were found in the following variables: grip strength, from 18.70 (17.98–19.23) at baseline to 21.57 (20.67–23.16) kg (*p* = 0.002); gait speed, from 0.95 (0.81–1.18) at baseline to 1.34 (1.20–1.47) m/s (*p* = 0.003); and hip circumference, from 99.75 (94.75–110.37) at baseline to 97.65 (93.92–109.50) cm (*p* = 0.023). Other measurements that appeared without changes were appendicular skeletal muscle mass, from 21.17 (18.58–22.33) at baseline to 20.77 (18.31–22.39) kg (*p* = 0.875), and the appendicular skeletal muscle mass index, from 8.64 (8.08–9.35) at baseline to 8.81 (7.91–9.38) kg/m^2^ (*p* = 0.875) after the intervention. Conclusions: The three-month multicomponent intervention in women at risk of sarcopenia improved their grip strength and gait speed.

## 1. Introduction

Aging is a universal process characterized by physiological changes in the human body that can jeopardize and deteriorate a person’s health. Among these changes is the modification of body composition, with an increase in body fat while the musculoskeletal mass decreases. The decrease in muscular tissue can cause predisposition to multiple diseases; among them is sarcopenia, characterized by a generalized loss in musculoskeletal mass, resulting in compromised muscle performance and functionality [1]. Since 2016, sarcopenia has been considered a disease (ICD-10-CM M62.84), and the European Working Group on Sarcopenia in Older People 2 (EWGSOP2) has proposed the following diagnostic algorithm for women: (1) a decrease in grip strength < 16 kg, (2) a decrease in the appendicular skeletal muscle mass (ASMM) < 15 kg or ASMM index (ASMMI) < 5.5 kg/m^2^, and (3) a reduction in walking speed ≤ 0.80 m/s, which determines the severity of the disease [1,2,3,4].

Musculoskeletal mass undergoes anatomical and functional changes throughout aging due to declining levels of testosterone and estrogen [5]. However, additional factors, such as chronic degenerative diseases, nutritional burden, and physical inactivity, may contribute to musculoskeletal mass loss. Chronic degenerative diseases are associated with a deleterious inflammatory cascade characterized by increased reactive oxygen species production, decreased numbers of satellite cells, and the presence of a nutritional burden; this latter factor might be a consequence of gastrointestinal changes, the loss of teeth, or cognitive impairment [6,7]. On the other hand, physical activity has been described as a protective factor that prevents muscle mass loss due to a promotion in protein synthesis and the prevention of fat infiltration to muscle cells [8,9].

The prevalence of sarcopenia varies over time due to the use of newer evaluation algorithms, as well as the lack of standardization of cutoff points, which was evident in a recent meta-analysis that reported a broad range of sarcopenia prevalence (from 10.0% to 27.0%) among adults >60 years of age [10]. Additional studies have shown an increase in prevalence from 26.3% to 40.0% [11]. In Mexico, the reported national prevalence is 13.3%. However, the prevalence varies according to age, with 26.3% in the 60–70 age range, 40.1% in the 70–79 range, and 33.6% in adults aged 80 or older. Furthermore, women who live in urban areas are at a higher risk for the development of sarcopenia [12].

Projections made by the World Health Organization (WHO) for the year 2050 predict that the older population will increase significantly worldwide [13], which may contribute to an increased prevalence of sarcopenia. Due to the increase in the risk of death, poor hospitalization outcomes, dependency, and disability associated with sarcopenia, it is of utmost importance to develop interventions that hinder the development and progression of this disease [14,15]. Physical activity is one of the best known non-pharmacological treatment alternatives due to its multiple health benefits; furthermore, by delaying muscle atrophy, it can aid in the prevention of sarcopenia associated with the natural aging process [16]. Additional benefits could be obtained by implementing multicomponent interventions (including resistance, flexibility, aerobic, and balance training) to maintain functionality [17].

Finally, scientific evidence has demonstrated that a combination of physical activity, an adequate diet, and supplementation could be a better strategy for treating sarcopenia compared to each intervention alone [18]. It has been shown that protein intake is decreased in older adults compared to younger adults, probably due to appetite reduction, anabolic resistance, medical conditions, and/or reduced economic resources. The daily recommended protein intake for older adults is 1.2–1.5 g per kilogram of weight, which will ensure a positive nitrogen balance, improve protein synthesis, and potentiate the effects of training [19,20]. However, protein sources should be provided by food, since protein-based supplements have shown discrepancies in terms of results [21].

The older population is usually heterogeneous, and each generation differs from the previous; therefore, the design of a sustainable strategy that is adaptable to heterogenous populations and can contribute to musculoskeletal health is of significance. In such a task, dancing has appeared as an attractive intervention, since it represents a social activity that has been part of human history for centuries and is a form of exercise that is adaptable to a person’s specific needs, including cultural and physical limitations. Current research focuses on studying the cognitive and visuospatial effects related to this activity, with interesting findings related to improvements in motivation, some memory aspects, and social cognition, as well as a reduction in distress [22,23]. Dancing can benefit the individual’s physical abilities, as it improves flexibility, resistance, and balance [24]; it could also be an option for improving grip strength, walking speed, and muscle thickness [25]. However, the effect of dancing in preventing sarcopenia still needs to be addressed appropriately, because its effects on ASMM have not been addressed. For this reason, this study aimed to evaluate a multicomponent intervention that consisted of a combination of dancing, resistance exercise, and nutritional education to prevent sarcopenia in women at risk of sarcopenia by assessing improvements in strength, ASMM, and gait speed.

## 2. Materials and Methods

### 2.1. Study Design

This study was a quasi-experimental multicomponent pilot study with measurements before and after, under a self-controlled design, which was approved by the Research and Ethics Committee of the Gynecology–Obstetrics Hospital of the Mexican Institute of Social Security (IMSS) at Western National Medical Center, located in Jalisco, Mexico (R-2019-1310-040), and registered on Clinical Trials as NCT06038500 (14 September 2023). The intervention was carried out in Biomedical Research Unit 02, attached to the Specialties Hospital Mexican Institute of Social Security, from July to September 2019. The study followed all principles of ethical standards.

### 2.2. Participant Eligibility

Our study recruited women via simple random sampling from the database of patients who regularly attend the climacteric clinic, and they were enrolled after providing written informed consent. The selection criteria were as follows: being a biological woman, aged 55–75 years, with current public health insurance and preserved physical and mental capacities (verified through data from the medical records), and at a risk of sarcopenia (i.e., a reduction in grip strength, with preserved ASMM and walking speed) [1]. The proposed criteria for this study were a low grip strength of <20.99 kg (this cutoff was validated for the Mexican population) [26], an ASMMI > 5.5 kg/m^2^, and a walking speed of >0.8 m/s. The exclusion criteria were related to conditions that could affect at least one of the following: (1) bioelectric activity (metal osteosynthesis systems or devices; pacemakers; defibrillators), (2) body composition (history of cancer, stroke, uncontrolled chronic degenerative diseases, risk of malnutrition, respiratory insufficiency, asthma or chronic obstructive pulmonary disease, hypothyroidism, hyperthyroidism, diagnosis of cognitive impairment made by a doctor, depression, hospitalization in the previous year), (3) limitations in carrying out the basic activities of daily living, e.g., balance disorders, (4) medical contraindications for physical activity, and (5) belonging to another physical activity group.

### 2.3. Multicomponent Intervention Approach

Our approach included a total of 24 sessions delivered over a 12-week period (2 sessions per week); the intervention consisted of a combination of resistance exercises, dancing sessions, and nutritional education. The four resistance exercises for the upper and lower extremities were biceps curls, triceps kickbacks, seated rows, lateral arm raises, squats, lateral walks, deadlifts, and calf raises. A low-intensity resistance band from 4 to 15 kg was used, with a length of 1.22 m (UltraSports™); only this type of band was used. No changes were made to the elastic band—only the volume (i.e., sets and repetitions) was adjusted—and the rest time between sets was 60–120 s. The workload in the first month consisted of two sets of 10 repetitions each, the second month was four sets of 8 repetitions, and the third month was four sets of 15 repetitions. Regarding dancing, the styles practiced throughout the program were salsa, merengue, rock and roll, cumbia, and cumbia norteña. Each session (dance + resistance exercise) was estimated to generate a caloric expenditure of 270.85 calories, corresponding to 3.5 and 4.5 METs per activity, according to the Compendium of Physical Activities [27]. The resistance exercises and the dance sessions began with a warm-up and stretching session, continued with walking, joint mobility exercises, and static flexibility (10 min), followed by the resistance exercises (approximately 30 min) and dancing practice (approximately 30 min), and ended with a cooling-down session, with movements focused on flexibility (10 min).

Lastly, nutritional education was delivered to the participants through group sessions occurring once a week, with a duration of 20 min; the sessions were conducted by a nutrition expert, covering topics related to nutrition, hydration, carbohydrates, protein, fats, fiber, dairy and non-dairy milk, probiotics and prebiotics, antioxidants, the recommended use of sugar and sweeteners, the importance of calcium and vitamin D, and the recommended diet for the maintenance of skeletal muscle mass, as suggested by the American College of Sports Medicine [28]. The nutritional education sessions were held after the exercise session, and the topics were reinforced by take-home brochures.

### 2.4. Outcome Measurements

The following were taken as indicators of sarcopenia: (1) Grip strength, which was evaluated using the JAMAR Plus+ Patterson Medical^®^ digital dynamometer, using the technique proposed by the American Society of Hand Therapists [29]. The calculation included the average of all attempts performed on each hand. (2) ASMM, which was calculated with the Yamada’s validated formula for multi-frequency devices using impedance values (Z) (Equation (1)) [30]. The ASMMI was obtained from ASMM (kg) divided between height^2^.

Formula for estimating ASMM proposed by Yamada et al.:

ASMM_kg_ = [((0.6144 × (Ht^2^/Z_50_)) + (−36.61 × (Z_250_/Z_5_)) + (−9332 × (1/Z_50_)) + 37.91)] (1)

Data for variable calculation were obtained using the TANITA MC 780 (Tanita, Tokyo, Japan) bioimpedance device, with a standing posture and eight electrodes. The bioimpedance test also provided body composition data such as the weight, body mass index (BMI), fat percentage, fat mass, fat-free mass, visceral fat, water percentage, and intracellular and extracellular fluids. For the basal and final measurements, the participants were asked to present after an eight-hour fast, including abstention from fluids; all metallic objects were removed before the bioimpedance measurements. (3) Gait speed, which was evaluated with a stopwatch to estimate the time after acceleration and before deceleration for a total distance of 4 m. The gait speed per meter was determined by dividing the distance walked by the time recorded. The study measurements were obtained in the morning at 8:00 a.m., one day before the intervention for basal measurements, and the day after the end of the final intervention for post-intervention parameters, after fasting with no water intake.

Anthropometric measurements, as stipulated by the ISAK (International Society for the Advancement of Kinanthropometry) protocol, were also considered [31]. Height was measured at the start of the evaluation using the Seca 213 stadiometer (Seca, Chino, CA, USA) without using footwear. A Lufkin anthropometric tape measure (NutriActiva, Minneapolis, MN, USA) was employed for the mid-arm, waist, hip, and calf measurements.

#### Other Clinical Variables

Obtained from the medical records, we registered information related to current pharmacological interventions, as well as the presence of specific conditions classified as gastrointestinal (e.g., colitis, diverticulitis, gastritis, reflux, constipation), cardiometabolic (e.g., diabetes, hypertension, dyslipidemia), and others (e.g., human papillomavirus, insomnia); the comorbidity assessment relied on the Charlson index [32]. The previous physical activity was evaluated with an affirmative response. Finally, polypharmacy was defined as the use of ≥5 drugs [33].

### 2.5. Compliance

Adherence to the program relied on an attendance list. Attendance < 80% was taken as an elimination criterion.

### 2.6. Statistical Analysis

The sample size calculation for a straightforward group, with a total of 14 participants, was based on the data from a previous study, with an alpha level of 0.05 and a beta level of 0.20 [34]. A descriptive analysis of the study participants’ sociodemographic characteristics and baseline measurements was performed. Qualitative data are presented as frequencies and percentages. The quantitative variables are described as medians and percentiles (25th–75th). The normal distribution of the data was analyzed using the Shapiro–Wilk test, the normality test value for sarcopenia components was <0.050, and the Wilcoxon test was used on non-parametric data to evaluate the effects on the study variables. A (*p*) value < 0.050 was considered statistically significant. The data were analyzed using the IBM SPSS version 23 statistical package.

## 3. Results

### 3.1. Participant Selection

**Figure 1:**
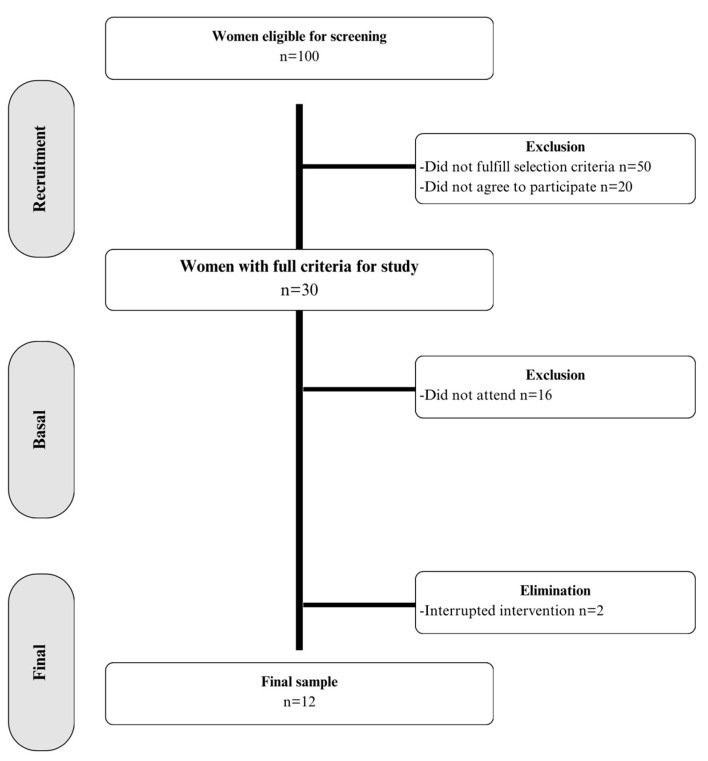
Sample eligibility flowchart for the study.

Figure 1 shows a flow diagram providing details related to the screening, enrolment, follow-up, and analysis of patients. Participants were selected from a cohort of 100 women who regularly attended the climacteric clinic. Primarily, they were verbally invited to carry out the examination. Even though 50 women met the inclusion criteria, only 30 were interested in participating; the primary reason for not being interested was difficulties in accessing transportation to the health center. Of those who were interested, only 14 started the intervention, and 2 participants withdrew during the study (after 6 and 10 sessions, respectively) for reasons unrelated to the study, such as having to care for a relative or wanting to spend more time with their partner. A comparative intention-to-treat analysis between the baseline and withdrawal measurements in the group that completed the study showed nonsignificant differences. No adverse effects occurred in any of the participants.

### 3.2. Sample Size and Group Characteristics

A total of 12 women completed the study, with a post hoc power of 73.1%. Regarding compliance, only 83.3% (n = 10) of the women achieved 100.0% attendance to sessions, and 16.7% (n = 2) of the women had an attendance percentage of 87.5%. Sociodemographic data such as the health status and lifestyle characteristics are shown in Table 1. An age of 55–65 years was recorded in 83.4% of the participating women. The highest level of education among the participants was the equivalent of senior high (66.7%). Most of the women were married (41.7%), and 75.0% were homemakers; the remaining 25.0% carried out informal work activities (25.0%)

Regarding health conditions, gastrointestinal diseases were most common among the participants (74.7%). Polypharmacy was present in only 25.0% of the sample. Concerning lifestyles, a history of physical activity prior to the study was reported in 41.7% of the women; the main type of activity described was up to 30 min of walking, with 60.0% of them performing the activity more than 3 days per week and only two participants performing this activity one to two times per week (40.0%). Regarding the indicators of sarcopenia, Table 2 shows how the participating women only had a reduction in grip strength according to the criteria established for this study (grip strength < 20.99 kg), but not in the ASMMI, ASMM, or gait speed, which meant they satisfied the criteria for sarcopenia risk.

### 3.3. Effects of the Multicomponent Intervention

**Figure 2:**
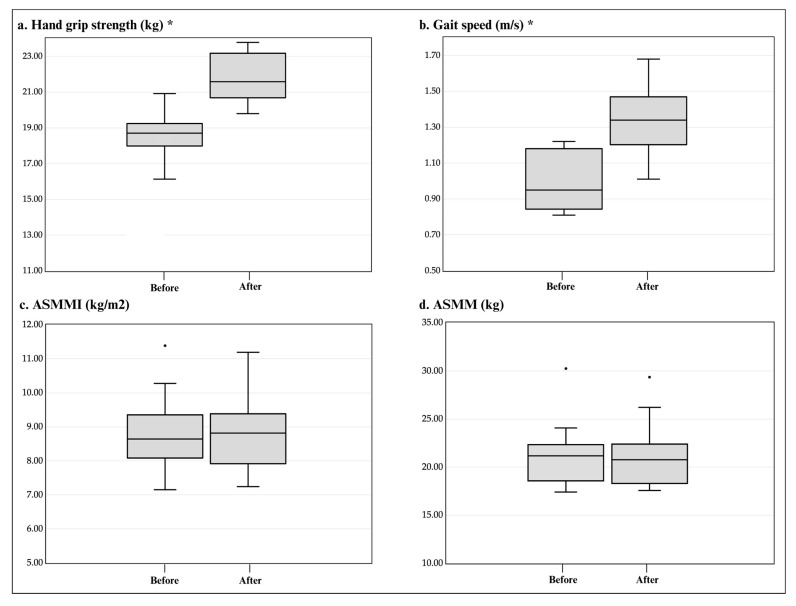
Boxplots before and after changes in the sarcopenia components. (**a**) Changes in grip strength from baseline to post-intervention. (**b**) Changes in gait speed from baseline to post-intervention. (**c**,**d**) Changes in ASMMI and ASMM, respectively, from baseline to post-intervention. The box represents the 25th and 75th percentiles, and the line represents the median. The circles represent outliers, and the asterisks in the boxplots for hand strength and gait speed indicate statistically significant differences between the basal and post-intervention measurements (Wilcoxon test). ASMMI: appendicular skeletal muscle mass index, ASMM: appendicular skeletal muscle mass, kg: kilograms, m: meters, s: seconds.

Three months after beginning our study, changes in sarcopenia indicators occurred. The first significant effect was on the grip strength, which went from a baseline measurement of 18.70 (17.98–19.23) kg to the post-test measurement of 21.57 (20.67–23.16) kg (*p* = 0.002); this value represents a change in muscle functionality status to no longer being at risk of sarcopenia (Figure 2a). Similarly, the participants’ gait speed showed a significant change, increasing from a baseline value of 0.95 (0.81–1.18) m/s to a final measurement of 1.34 (1.20–1.47) m/s (*p* = 0.003) (Figure 2b), thus revealing improved muscle performance. The change in ASMMI remained insignificant, from 8.64 (8.08–9.35) kg/m2 to 8.81 (7.91–9.38) kg/m^2^ (*p* = 0.875) (Figure 2c). In addition, the ASMM did not present statistical changes from the baseline to the end of the study, going from 21.17 (18.58–22.33) kg to 20.77 (18.31–22.39) kg (*p* = 0.875) (Figure 2d).

Table 3 shows other body composition measurements, including a significant reduction in the total hip circumference (−2.10 cm, *p* = 0.041). Although weight also decreased, this was statically insignificant (−1.20 kg, *p* = 0.108). In addition, body fat and visceral fat showed no significant reductions throughout the intervention; the average loss of adipose tissue was 1.50 kg (*p* = 0.182), and the change in the level of visceral fat was 0.50 points (*p* = 0.157). Finally, the percentages of intracellular and extracellular water, along with the BMI, remained constant throughout the study.

## 4. Discussion

This study aimed to evaluate, in an exploratory manner, the effects of a combined intervention defined as a multicomponent approach that included resistance exercises, dancing, and nutritional education in a group of women at risk of sarcopenia. This combined intervention, to the best of the authors’ knowledge, has not been tested before. Resistance exercise is the most recommended activity for treating sarcopenia, as it favors the growth and maintenance of ASSM [35]. However, the combination of different exercise variants, such as aerobic and anaerobic exercises, may be an adequate strategy for improving sarcopenia, since it contributes to improving insulin resistance and mitochondrial function, as well as increasing satellite cells and protein synthesis [36,37].

Our results demonstrate that the proposed intervention showed a possible benefit in improving strength and gait speed in women who are at risk of sarcopenia. This study provides a preliminary assessment of benefits that should be evaluated under a larger definitive trial. Using a multicomponent approach with combined interventions, observable beneficial effects can be obtained not only in performance [38] but also in grip strength and ASSM, with the addition of essential amino acids (which have also proven useful), after three months [39]. Performing resistance exercises two or three times per week can improve leg and chest strength in older adults [40], depending on the level of intensity of the performed activity [39]. However, the intensity, volume, and frequency could affect the final effects. In this study, exercise intensity was not measured through a stress test or by heart rate, but this intervention was delivered through progressive changes in workload that could facilitate a significant increase in grip strength, even though the intervention had a duration of only three months, which represents the minimum duration previously reported to obtain benefits in patients with sarcopenia [36].

Implementing a multicomponent approach may be an optimal strategy for improving musculoskeletal health, as combining different activities could potentiate their effects. Because exercise is the primary non-pharmacological treatment option for sarcopenia, resistance band training could be an option for maintaining ASMM [41]. Hence, the use of resistance bands has proven to have an advantage in offering a greater range of motion and variable resistance; moreover, it is safe, easy, and affordable for older people [25].

Although dancing could increase ASMM, the effect is not entirely known; in this study, a reduction in ASMM was found, but tissue loss was not significant. On the other hand, a significant effect on muscle strength has been shown in a previous study [42]. Other authors have shown that this activity alone may not be enough to improve strength but is sufficient to improve walking speed [43], even to a greater extent than the resistance exercise itself [44]. In addition, variations in the type of dancing, the studied population, the duration of the intervention, and the conditions associated with the exercise (such as clothing, which can sometimes function as extra weight for a person while dancing) can be considered as important variables in addressing the benefits of the exposure [42]. Therefore, the suggestion is to complement dancing with resistance exercise to improve ASMM, because both approaches can affect strength and ASMM when implemented separately [23,25,36].

Another aspect to consider is the nutritional status and supplementation, as these could be determining factors in a multicomponent approach. Kim et al. [41] showed increased ASMM through a multicomponent approach that included two weekly sessions for three months; it is worth noting that the participants received nutritional supplementation. Unlike the previous study, our group received no nutritional supplementation, only nutritional education. Supplements can help to ensure adequate protein and calorie intake, which can contribute to maintaining and improving muscle. In our study, even in the absence of supplement intake, we could identify a significant change in muscle strength; however, muscle strength is independent of ASMM, for the increase in strength is not always accompanied by an increase in ASMM [45]. Furthermore, the type of device used to evaluate this tissue (impedance) can modify this value. Despite being a practical, fast, and non-invasive tool, it does not allow for the identification of changes in the muscular architecture, unlike other procedures such as magnetic resonance imaging, computed tomography, ultrasound, or muscle biopsy.

Our failure to find a significant increase in ASMM and ASMMI may have been due to insufficient muscle stimulation, as we found differences in the design compared to previously published papers. One difference was the short duration of the intervention, although recent studies carried out with resistance bands over a six-month program did not report significant changes in ASMM either [46]. Another difference is the fact that we used a single type of resistance, with one type of elastic band, unlike other studies that implemented different types of resistance [25]. A third difference is the instrument used to measure ASMM, since favorable changes have been reported after the conduction of imaging techniques such as ultrasound [23]. In the same way, previous studies have implemented variations in interventions related to the volume, the level of intensity, and the material used, with favorable effects on ASMM [36,47]. Finally, the unstoppable aging process also favors anabolic resistance, fat infiltration into muscle, and an increase in the total fat mass. One of the limitations of our study that could have influenced the lack of changes in ASMM and ASMMI was the inability to implement the strict control of the daily caloric/protein intake in participants or evaluate the perception of effort.

Another point is the population studied, since, according to the BMI results, most individuals from the study group were overweight or obese, so the different circumference values exceeded the values considered to be within normal parameters, translating into a greater risk for the development of chronic non-communicable diseases. The participating women reflected the high prevalence of overweight and obesity that has been observed recently in all age groups in the Mexican population [48].

Data from the National Institute of Statistics and Geography in Mexico (INEGI) show that 68.7% of women over 55 years of age do not perform any physical activity during their free time [49]; 58.3% of our sample was sedentary, as they did not perform any physical activity, and none of the participants performed resistance exercises before the study. The adaptation mechanisms and the supercompensation theory could also explain the changes observed with this intervention.

Finally, regarding body composition, no changes were found in the fat percentage, fat mass, and visceral fat variables, consistent with the findings of other studies [50,51]. However, this pilot study did not aim to improve adiposity levels; a more extensive intervention might be needed to change body composition [36]. For example, an 18-month dancing study revealed a significant effect on adipose tissue [52]. Apart from the total duration of the intervention, the number of sessions per week is also essential, as a minimum number of sessions has been recommended; other studies have reported up to five days of physical activity per week [40,52].

The limitations of the present study include its small sample size, the lack of a control group, and the lack of nutritional supplementation. In addition, adherence to the nutrition program and participants’ diet changes were not evaluated. However, this is a pilot study, with favorable results for a change in two of the three indicators for sarcopenia in a sedentary group with a short intervention. Additional studies that include dietary changes and the use of supplementation will be needed in order to reinforce our findings and define a possible prevention strategy for sarcopenia.

## 5. Conclusions

This study provides preliminary results demonstrating that a three-month multicomponent preventive intervention, consisting of a combination of resistance exercise, dance, and nutritional education, improved grip strength and gait speed in women at risk of sarcopenia.

## Figures and Tables

**Table 1:** Sociodemographic and health characteristics of the participants.

Sociodemographic Characteristics (n = 12)	n (%)
**Age (years)**	
55–65	10 (83.4)
66–75	2 (16.6)
**Education level**	
Elementary	4 (33.3)
High school	8 (66.7)
**Marital status**	
Married	5 (41.7)
Single	3 (25.0)
Divorced	4 (33.3)
**Occupation**	
Housewife	9 (75.0)
Employed	3 (25.0)
**Home**	
Living alone	7 (58.3)
Family living	5 (41.7)
**Health status characteristics (n = 12)**	
**Reported diseases**	
Gastrointestinal	9 (74.7)
Cardiometabolic	8 (66.7)
Other	4 (33.3)
**Polypharmacy**	
No	9 (75.0)
Yes	3 (25.0)
**Comorbidity**	
Absence of comorbidity	10 (83.3)
Low comorbidity	2 (16.7)

Direct source.

**Table 2:** Baseline components of sarcopenia.

Functionality and Performance Markers, n = 12	Basal Median (25th–75th)
Grip strength (kg)	18.70 (17.98–19.23)
ASMMI (kg/m^2^)	8.64 (8.08–9.35)
ASMM (kg)	21.17 (18.58–22.33)
Gait speed (m/s)	0.95 (0.81–1.18)

Direct source; 25th–75th percentiles, ASMMI: appendicular skeletal muscle mass index, ASMM: appendicular skeletal muscle mass, kg: kilograms, m: meters, s: seconds.

**Table 3:** Elements of body components, pre-test vs. post-test.

Variable, n = 12		Pre-Test Median (25th–75th)	Post-Test Median (25th–75th)	*p*	∆
**Anthropometric**	Height (m)	1.55 (1.53–1.56)	1.55 (1.53–1.56)	1.000	0.00
	Weight (kg)	61.85 (54.47–75.85)	60.65 (54.47–75.85)	0.108	−1.20
**Circumference**	Mean arm circumference (cm)	30.70 (27.62–34.87)	30.50 (27.27–33.95)	0.444	−0.20
	Waist (cm)	89.10 (81.37–99.25)	89.75 (78.75–97.12)	0.328	0.65
	Hip (cm)	99.75 (94.75–110.37)	97.65 (93.92–109.50)	0.023 *	−2.10
	Calf (cm)	34.50 (31.87–37.15)	33.65 (32.25–36.52)	0.554	−0.85
**Body composition**	Mass fat (%)	36.55 (32.77–36.55)	35.35 (32.52–37.70)	0.308	−1.20
	Mass fat (kg)	22.60 (20.20–29.82)	21.10 (17.70–21.10)	0.182	−1.50
	Fat-free mass (kg)	39.65 (35.62–45.50)	40.15 (35.82–45.30)	0.610	0.50
	Visceral fat level (scores)	9.00 (8.00–9.00)	8.50 (7.00–8.50)	0.157	−0.50
	Percentage of water (%)	44.30 (43.52–46.27)	45.60 (43.97–47.67)	0.182	1.30
	Intracellular water (L)	14.90 (13.45–17)	15.10 (13.37–16.85)	0.326	0.20
	Extracellular water (L)	13.10 (11.90–15.17)	13.15 (11.85–15.27)	1.000	0.05
	BMI (kg/m^2^)	25.25 (23.36–25.25)	24.84 (22.73–31.76)	0.099	−0.41

Direct source: 25th–75th percentiles; ∆: delta score (post-test data minus pre-test data); * significant according to the Wilcoxon test. BMI: body mass index, m: meters, kg: kilograms, cm: centimeters, L: liter, %: percentage.

## Data Availability

Data are contained within the article.
